# Evidence for I_2_ loss from the perovskite–gas interface upon light-induced halide segregation†

**DOI:** 10.1039/d4sc08092k

**Published:** 2025-04-28

**Authors:** Michael Lee, Julian A. Vigil, Zhiqiao Jiang, Hemamala I. Karunadasa

**Affiliations:** a Department of Chemistry, Stanford University Stanford California 94305 USA hemamala@stanford.edu; b Department of Chemical Engineering, Stanford University Stanford California 94305 USA; c Department of Materials Science and Engineering, Stanford University Stanford California 94305 USA; d Stanford Institute for Materials and Energy Sciences, SLAC National Laboratory Menlo Park CA 94025 USA

## Abstract

Sunlight-induced halide segregation in (CH_3_NH_3_)Pb(Br_*x*_I_1−*x*_)_3_ (1 > *x* > 0.2), which limits obtainable voltages from solar cells with these perovskite absorbers, reverses upon resting in the dark. However, sustained illumination at *ca.* 1 sun opens a new decomposition pathway, leading to irreversible I_2_ loss in an open system. We conclusively show I_2_ off-gassing from halide-segregated (CH_3_NH_3_)Pb(Br_0.75_I_0.25_)_3_ by trapping gaseous I_2_ and tracking the electronic conductivity of the perovskite, which increases from electron-doping as iodides are oxidized to iodine. Importantly, we show that this reaction occurs across the perovskite-air solid–gas interface, without confounding effects from solvent or reactive solid interfaces. This characterization was conducted under a nitrogen atmosphere, avoiding vacuum- and oxygen-driven I_2_ loss pathways. Consistent observations of I_2_ loss upon light-soaking CsPb(Br_0.75_I_0.25_)_3_ films show that this reaction is intrinsic to the inorganic framework. We propose that the disruption of iodide-rich domains in the halide-segregated films through I_2_ loss can masquerade as a light-induced healing or apparent remixing of the segregated film, when in fact it leads to irreversible decomposition. Although I_2_ off-gassing is less likely in bromide-rich solid solutions, light-induced halide segregation brings the iodides into proximity and forms electronic states that are energetically poised to trap and accumulate holes, providing a driving force for I_2_ loss. Thus, even bromide-rich mixed-halide perovskite absorbers will benefit from I_2_-impermeable encapsulation for long-term stability.

## Abstract 2

Sunlight-induced halide segregation in (CH_3_NH_3_)Pb(Br_*x*_I_1−*x*_)_3_ (1 > *x* > 0.2) reverses upon resting in the dark, but sustained illumination at ambient conditions leads to irreversible I_2_ loss in an open system.

## Graphical abstract

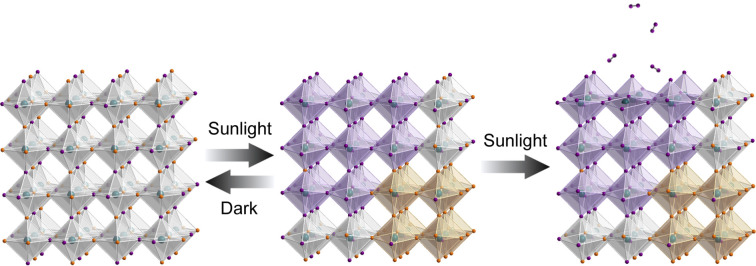

## Introduction

Lead-halide perovskites have emerged as a leading class of absorber materials for thin-film photovoltaic devices.^1–3^ At the forefront of applications is the use of perovskites in tandem solar cells,^4^ where a higher-bandgap perovskite accompanies a lower-bandgap absorber such as silicon,^5,6^ which have demonstrated impressive power conversion efficiencies well in excess of 30%.^7,8^ The ideal bandgap for an absorber used in tandem with silicon is 1.7–1.8 eV—^9,10^higher than the 1.5–1.6 eV bandgap common for lead-iodide perovskites^11,12^—which demands bandgap tunability from the halide perovskites. Given that the valence and conduction bands of the lead-halide perovskites have dominant halide character,^13,14^ bandgap control is easily achieved by tuning the halide composition.^15^ For example, alloying bromide ions into a lead-iodide perovskite increases its bandgap to the ideal range for a tandem solar cell. However, even after tuning the halide composition to reach optimal bandgaps, the expected device-level change—in particular, an increased open-circuit voltage compared to those produced by lead-iodide perovskite absorbers—did not follow.^15^ The demonstration that photoinduced halide segregation, where iodide- and bromide-rich regions form upon illumination of the mixed-halide perovskite absorber,^16^ caused the diminished voltage would prove massively consequential in device design. Indeed, the majority of recent high-efficiency perovskite/Si tandem cells are designed with halide segregation in mind.^7,8,17,18^

Although mixed-halide perovskites, such as (MA)Pb(Br_*x*_I_1−*x*_)_3_ (MA = CH_3_NH_3_^+^; 1 > *x* > 0.2), initially show the expected band-edge photoluminescence (PL), upon moderate visible-light soaking (15 mW cm^−2^ for more than one minute) this PL peak diminishes to be replaced by a lower-energy PL peak at *ca.* 1.7 eV.^16^ This observation, along with many others,^16,19,20^ could be explained through the formation of iodide- and bromide-rich domains, with bandgaps that are smaller and larger, respectively, than those of the unsegregated mixed-halide perovskite film. Therefore, the smaller bandgaps of the iodide-rich domains trap photogenerated carriers that form throughout the perovskite, affording the lower-energy PL.^21^ As such, a PL peak that redshifts to *ca.* 1.7 eV has been used as a diagnostic for light-induced halide segregation. Notably, this light-induced halide segregation appeared to be reversible over several cycles of illumination and resting in the dark for several minutes.^16^

Amidst the surge of research surrounding halide segregation and potential methods to mitigate it^22,23^ came recent claims of halide re-mixing in halide-segregated perovskites—one in (MA)Pb(Br_0.8_I_0.2_)_3_ micron-sized crystals^24^ and the other in CsPb(Br_0.6_I_0.4_)_3_ polycrystalline films^25^—after exposure to higher-intensity illumination. These reports showed that the lower-energy PL (characteristic of the iodide-rich domains in the halide-segregated perovskite) could be replaced by the higher-energy PL (reminiscent of the original mixed-halide perovskite) if the perovskites were exposed to higher-intensity light (1.5 W cm^−2^ for 30 minutes for CsPb(Br_0.6_I_0.4_)_3_ films; 200 W cm^−2^ for 20 s for (MA)Pb(Br_0.8_I_0.2_)_3_ crystals), suggesting intriguing possibilities of “healing” or halide re-mixing through additional light-soaking. However, although not mentioned in the report, a close examination of the optical spectra of purportedly self-healed (MA)Pb(Br_0.8_I_0.2_)_3_ crystals^24^ show a slightly blueshifted PL compared to that of the original perovskite, consistent with the overall loss of iodide and resulting in a larger bandgap.

Several other studies have noted the same loss of low-energy PL in halide-segregated perovskite films under high-intensity illuminations. An older study^26^ observed this in (MA)Pb(Br_2.5_I_0.5_) films and used controlled atmosphere studies to attribute this PL loss to O_2_-mediated decomposition of the iodide-rich domains by comparison to known decomposition pathways of (MA)PbI_3_.^27^ Another group of studies argue for a photothermal halide remixing process that competes with halide segregation, based on PL spectra of polystyrene-encapsulated (MA)Pb(Br_*x*_I_1−*x*_)_3_ films (illuminated with a 473 nm continuous-wave laser at 16 W cm^−2^ for up to 200 s).^28,29^ This claim was supported by separate temperature-dependent studies indicating the presence of a competing process to halide segregation.^30^ These contradictory findings on the effects of light-soaking halide-segregated films, and the blueshifted PL of the purportedly “healed” perovskites, motivate further investigation of potential iodide loss from mixed-halide perovskites under high-intensity illumination.

Although I_2_ loss is most likely to be light-induced, other mechanisms can result in iodide loss from perovskites, especially in organic–inorganic compositions where HI evolution is possible. Iodine loss has particular implications for device stability, with the removal of iodide ions,^31^ the resulting iodide vacancies,^32^ and the corrosive I_2_ vapor^33,34^ all being connected with photodegradation of perovskite absorber layers. We therefore looked for clear evidence of I_2_ off-gassing from the perovskite-air interface—without the presence of solvent or oxygen—of mixed-halide perovskites under illumination.

## Prior work on light-induced halogen loss

X-site self-exchange is commonly observed in the ABX_3_ perovskite family. Oxide perovskites maintain a well-studied equilibrium between oxide sites in the crystal and oxygen gas,^35^ and there is growing evidence of an analogous equilibrium in the halide perovskites through eqn (1),^36,37^ where *X*_*X*_ is an occupied halide site, 
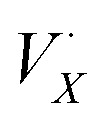
 a monocationic halogen vacancy, e^′^ an electron, and *X*_2_ the diatomic halogen gas.1
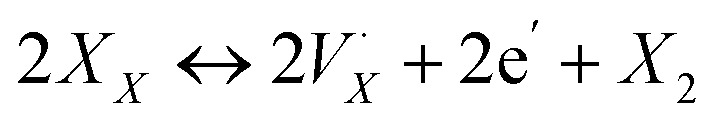


Both directions of the equilibrium in eqn (1) have been shown separately in halide perovskites, even without illumination or heating above ambient temperature. For example, the presence of I_2_ in the atmosphere was shown to p-dope polycrystalline (MA)PbI_3_ thin films (reverse reaction) through work-function measurements.^38^ Both the forward and reverse reactions were inferred from electronic conductivity measurements of a double perovskite single crystal Cs_2_AgTlBr_6_ through exposure to a Br_2_ atmosphere.^36^ The thermodynamics of the equilibrium was studied in a single crystal of the double perovskite Cs_2_SnI_6_; spontaneous I_2_ off-gassing near room temperature is enabled by a low enthalpy of reaction—breaking metal-iodide bonds and forming I_2_—and the very large entropy gain for this solid-to-gas reaction.^37^

Expecting illumination to play a role in halogen off-gassing from halide perovskites can also be justified. The valence-band maximum of most halide perovskites, where photogenerated holes accumulate, is primarily formed by halide p orbitals, leading to halide oxidation and subsequent loss as halogen gas.^13^ Indeed, halide oxidation under illumination has been studied for centuries, with photodecomposition of silver bromides and iodides to silver metal and halogen vapor serving as the basis of black-and-white photography since the 1830s.^39^ Time-resolved mass spectrometry and X-ray photoelectron spectroscopy measurements evidence I_2_ release from (MA)PbI_3_ under dynamic vacuum in the dark^40^ and under illumination.^40–42^ Further, I_2_ has been detected during illumination of (MA)PbI_3_ immersed in toluene, by identifying the I_2_ charge–transfer complex (I_2_·toluene) using solution-state absorbance measurements.^31^

Such I_2_ loss should be less likely in Br-rich mixed-iodide-bromide perovskites. However, light-induced I^0/−^ loss through the solid–liquid interface between these perovskites and organic solvents has been inferred from solution-state measurements of the formation of I_3_^−^ in dichloromethane (DCM) or I_2_·toluene in toluene, alongside a steadily increasing bandgap under continuous illumination. Here, we refer to the leaving ligand as I^0/−^, as the mediating solvent complicates the assignment of the species leaving the perovskite as I^−^ or I_2_, as described later. For example, films of mixed-halide perovskites (such as (MA)PbI_1.5_Br_1.5_ (ref. 43) or Cs_0.17_FA_0.83_PbI_1.5_Br_1.5_;^44^ FA = HC(NH_2_)_2_^+^) showed the expected light-induced halide segregation, immersing these films in DCM or toluene and irradiating them led to the formation of I_3_^−^ or I_2_·toluene, detected by solution-state absorbance measurements.^43,44^ The electrochemical injection of holes into mixed iodide-bromide perovskites was also shown to release I^0/−^ through UV-visible spectroscopic detection of I_3_^−^ in DCM.^45,46^

Overall, numerous prior studies suggest the oxidation of iodide in the perovskite to iodine, facilitated by light (or the accumulation of holes) and possibly solvent. However, the solvent–perovskite interface complicates the unambiguous identification of the species leaving the perovskite as I_2_. Although these experiments were performed in DCM or toluene, solvents in which halide perovskites do not visibly dissolve, these solvents can still crystallize halide perovskites and mediate reactions between halide perovskites and reagents, implying slight dissolution and re-precipitation at the perovskite surface.^47^ If even slight dissolution leads to the loss of I^−^ ions from the perovskite, then light or O_2_ can oxidize these free I^−^ ions in solution into I_2_ (or equivalently I_2_ + I^−^ = I_3_^−^), further driving dissolution through Le Chatelier's principle. From UV-visible absorption spectra, we can indeed infer the presence of iodide in DCM sealed with (MA)Pb(Br_0.75_I_0.25_)_3_ powder in the dark over 1 day (Fig. S1†). Further, DCM is also known to produce Cl˙ radicals upon irradiation, a strong oxidant that could react spontaneously with I^−^.^48–50^ Such decomposition pathways, involving dissolved iodides, are unlikely to occur in perovskites not immersed in a solvent; more importantly, these reactions are not representative of defect reactions that electronically dope the perovskite. Thus, the solid–air interface is most relevant when studying possible degradation during solar cell operation, particularly since doping of the perovskite can dramatically change the bulk electron transport properties.

Given the importance of identifying degradation pathways in perovskite compositions of great utility in high-efficiency tandem solar cells,^7,8^ we sought to build on the prior studies to show facile I_2_ loss through the perovskite-atmosphere interface in the absence of O_2_. To our knowledge, this work represents the first direct, conclusive evidence of sunlight-driven I_2_ off-gassing from a mixed iodide-bromide perovskite in realistic operating conditions (ambient pressure). To conclusively demonstrate illumination-driven I_2_ loss, we:

(1) used a solid chemical trap to detect I_2_ gas evolving from the perovskite into an O_2_-free atmosphere under simulated solar illumination, and compared the observations to those from heating the perovskite in the dark, and (2) showed that the electronic conductivity of the perovskite increases upon illumination, evidencing the n-type doping of the perovskite that accompanies I_2_ loss. Importantly, (2) distinguishes I_2_ off-gassing from I^−^ loss (as (MA)I or HI), where the latter would not electronically dope the perovskite.

## Detection of I_2_ evolving from the solid–gas interface

**Fig. 1:**
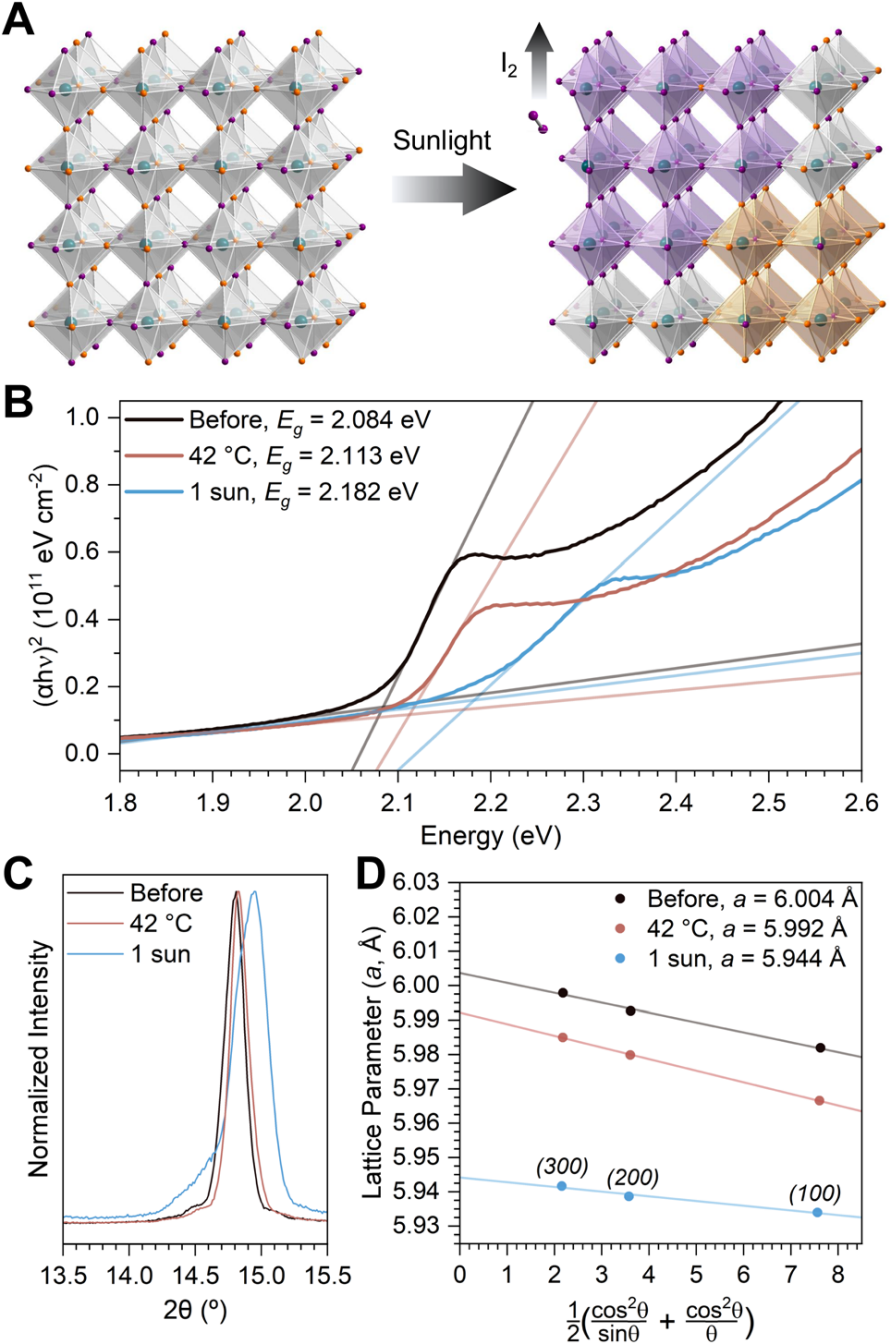
Evidence of average composition changes after illumination of a mixed iodide-bromide perovskite | (A) schematic illustration depicting the loss of I_2_ following illumination and halide segregation; octahedra comprising [PbI_6_]^4−^ and [PbBr_6_]^4−^ are shaded in violet and orange, respectively. Figure adapted with permission from ref. 51. (B) Tauc plots from optical transmission measurements of (MA)Pb(Br_0.75_I_0.25_)_3_ thin films following heating at *ca.* 42 °C (35 minutes; red trace) or illumination at 1 sun (30 minutes; blue trace); optical bandgaps (*E*_g_) obtained from linear-intersection fitting (see ESI†). (C) X-ray diffraction patterns showing the shift in the pseudo-cubic (100) Bragg reflection of the (MA)Pb(Br_0.75_I_0.25_)_3_ thin films following heating or illumination (as in 1B). (D) Pseudo-cubic lattice parameter, *a*, estimation using the Nelson–Riley method^52^ (see ESI†); points correspond to the fitted center (in 2*θ*) of the corresponding Bragg peaks (Fig. S2†).

To illustrate the effect of illumination on a mixed-halide perovskite, spin-cast thin films of (MA)Pb(Br_*x*_I_1−*x*_)_3_ were used as a representative model. An approximate halide composition of *x* = 0.75, based on the precursor solution (see Methods; ESI†), was chosen to provide sufficient bromide content for halide segregation. Each film was cut into two pieces and half of each film was illuminated under one sun (AM1.5G, 0.1 W cm^−2^ simulated) for 30 minutes in a glass chamber sealed in a nitrogen atmosphere. For direct comparison, the other half of the film was heated to *ca.* 42 °C (the highest temperature reached under illumination, Fig. S3†) for 35 minutes in an identical glass chamber sealed under nitrogen. The optical bandgap obtained from fitting absorption spectra show a clear blueshift, from 2.084 eV to 2.182 eV for the illuminated film, consistent with a more bromide-rich average composition (Fig. 1B). X-ray diffraction (XRD) measurements of the illuminated film reveal Bragg peaks systematically shifted to higher angles (Fig. 1C) and a decrease in fitted pseudo-cubic lattice parameter from 6.004 Å to 5.944 Å (Fig. 1D), commensurate with Br enrichment while preserving the overall crystal structure. Both signs of iodide loss seen for the illuminated film were observed in the heated films with smaller magnitudes of change (Fig. 1B and C).

Similar signs of iodide loss were also observed from CsPb(Br_*x*_I_1−*x*_)_3_ thin films of similar halide composition (*x* ∼0.75) that were subjected to the same heating and illumination conditions, although the effects were less pronounced compared to those of the hybrid perovskite. With light-soaking, the optical bandgap of the fitted UV-visible absorption spectra blueshifted from 2.174 eV to 2.196 eV, whereas the heated film showed a smaller shift in bandgap (Fig. S4†). The fitted pseudo-cubic lattice parameter extracted from XRD patterns also decreased from 5.899 Å to 5.877 Å in the light-soaked perovskite, whereas the heated film showed a smaller decrease (Fig. S5†). Because the same trends are seen in this inorganic analog, which is considered to be more stable,^25^ we posit that iodide loss is inherent to the metal-halide framework and not directly tied to reactivity with—or volatilization of—an organic cation, although interactions with the A-site cation may influence the reaction rate.

**Fig. 2:**
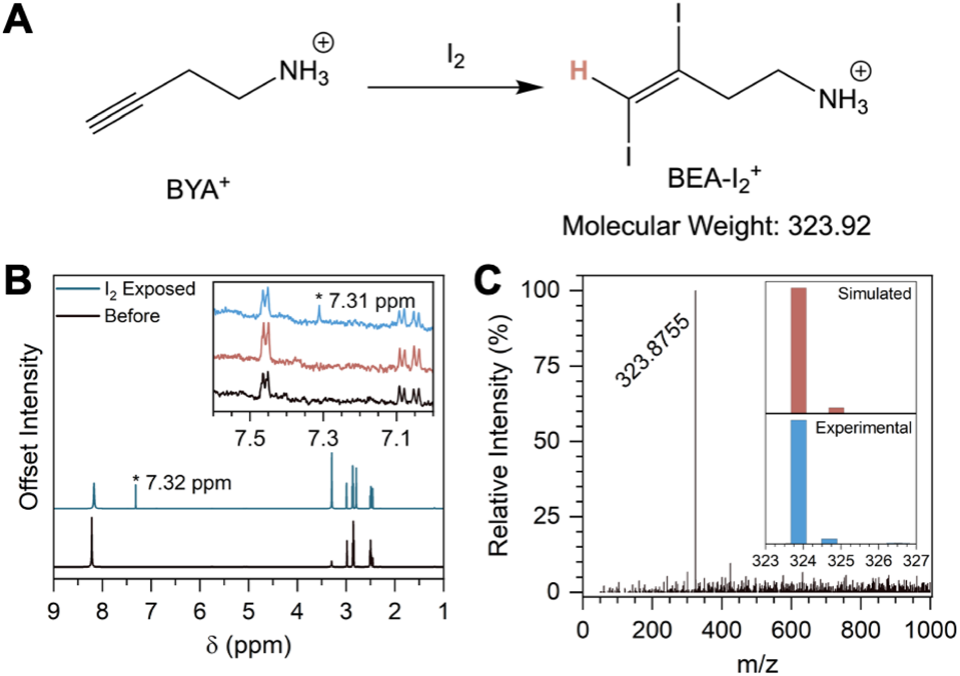
Direct detection of I_2_ off-gassing | (A) reaction scheme for the irreversible iodination of BYA^+^ to form BEA-I_2_^+^ (the chloride counter-ion is omitted for clarity and the vinylic hydrogen of BEA-I_2_^+^ is highlighted in red). (B) ^1^H-NMR spectra of solid (BYA)Cl dissolved in *d*_6_-DMSO (black trace) and of solid (BYA)Cl exposed to a saturated I_2_ atmosphere (40 °C, 16 h) and then dissolved in *d*_6_-DMSO (dark blue trace). The asterisk denotes the peak corresponding to the vinylic hydrogen of BEA-I_2_. The inset shows the reaction products (in DMSO) of solid (BYA)Cl after illumination (1 sun, 15 h; blue trace) or heating (45 °C, 15 h; red trace) in the presence of (MA)Pb(Br_0.75_I_0.25_)_3_ powder. (C) Mass spectrum of solid (BYA)Cl, which was exposed to 1 sun illumination in the presence of (MA)Pb(Br_0.75_I_0.25_)_3_ powder then dissolved in methanol; insets show the experimental and simulated peak corresponding to BEA-I_2_^+^ (calculated *m*/*z* = 323.87).

To assess whether these apparent changes in the average composition originate from I_2_ off-gassing, a bulk powder of (MA)Pb(Br_0.75_I_0.25_)_3_ was sealed in the presence of a chemical trap, but-3-yn-1-aminium (BYA^+^) chloride. Exposure of the alkyne BYA^+^ to I_2_ vapor causes spontaneous, irreversible iodination, yielding (*E*)-3,4-diiodobut-3-en-1-aminium (BEA-I_2_^+^; Fig. 2A). In contrast to detecting I_2_ dissolved in toluene or DCM, this reaction progresses spontaneously without direct contact between the perovskite and the solid BYA^+^ chloride salt ((BYA)Cl).

The black trace in Fig. 2B shows the ^1^H nuclear magnetic resonance (NMR) spectrum of (BYA)Cl in *d*_6_-DMSO. Upon exposing solid (BYA)Cl to I_2_ gas and then dissolving the solid in *d*_6_-DMSO, we see the appearance of a vinylic peak shifted to 7.3 ppm (blue trace), indicating the conversion of (BYA)Cl to (BEA-I_2_)Cl (Fig. S6†). This same peak is present in the NMR spectrum of solid (BYA)Cl that was first illuminated for 16 h in the presence of (MA)Pb(Br_*x*_I_1−*x*_)_3_ powder and then dissolved in *d*_6_-DMSO, directly showing the release of I_2_ vapor from the perovskite during illumination (Fig. 2B, inset). Analogous measurements done after heating to *ca.* 45 °C over the same period do not show a detectable peak at 7.3 ppm, suggesting the release of I_2_ does not originate from heating during illumination. In an accompanying mass spectrometry measurement of the same sample, an ion matching the *m*/*z* value for BEA-I_2_^+^ was obtained from the illuminated sample (Fig. 2C). No species with an *m*/*z* corresponding to any other addition products of the chemical trap were detected (*e.g.*, BEA-HI^+^, BEA-IBr^+^, BEA-Br_2_^+^). There was not enough BEA-I_2_^+^ to be detected by the same measurement in the sample that was heated in the dark, supporting the conclusions drawn from the NMR spectra (Fig. S7†).

## Electronic doping with loss of I_2_

Because iodide loss through I_2_ off-gassing as described in eqn (1) dopes the perovskite with excess electrons^36,37^—assuming the iodine vacancy defect is ionized—electronic conductivity changes are expected following illumination. Although intrinsic defect formation in most lead-halide perovskites is known to be charge-compensated,^53,54^ the Fermi level has been shown to shift down (becoming more p-type) in (MA)PbI_3_ films upon I_2_ exposure^38^ and to shift up (becoming more n-type) in CsPb(Br_*x*_I_1−*x*_)_3_ films upon I_2_ loss through extended UV illumination under vacuum.^55^ The change of an illuminated (MA)PbI_3_ film from p-type to n-type upon apparent iodine loss through a solvent, and the reverse effect upon exposing the film to an I_2_ atmosphere, has also been confirmed through Hall effect measurements.^31^

**Fig. 3:**
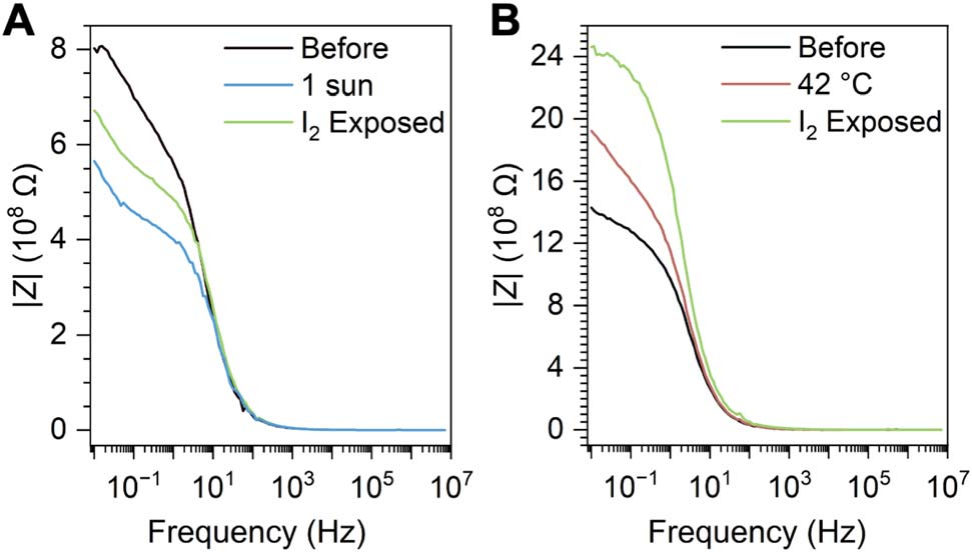
Distinguishing electrical doping in the illuminated mixed iodide-bromide perovskite | (A) Bode plots of (MA)Pb(Br_0.75_I_0.25_)_3_ films on interdigitated ITO substrates before (black trace) and after illumination (1 sun, 30 minutes; blue trace), and after subsequent I_2_ exposure (saturated atmosphere, 15 minutes; green trace). (B) Bode plots of films of (MA)Pb(Br_0.75_I_0.25_)_3_ before (black trace) and after heating (45 °C, 40 minutes; red trace) and after subsequent I_2_ exposure (saturated atmosphere, 15 minutes; green trace), showing a steadily increasing resistance. See main text for details. *Z* = electrochemical impedance.

Thin films of (MA)Pb(Br_*x*_I_1−*x*_)_3_ were spin-cast on interdigitated indium tin oxide electrodes to measure the impedance spectra of the perovskite films. Impedance spectra were collected between 7 MHz and 10 mHz; the bulk electrical resistance was evaluated from the low-frequency limit. The resistance of the film decreases following illumination (from 0.802 GΩ to 0.566 GΩ), consistent with I_2_ loss increasing the majority carrier (e^−^) concentration (Fig. 3A). Heating to 42 °C in the dark distinctly increases the resistance (from 1.43 GΩ to 1.92 GΩ), likely due to decomposition, further indicating that n-doping is a light-driven process (Fig. 3B). Subsequent exposure to I_2_ vapor in a nitrogen atmosphere for 20 minutes shows that the resistance of the illuminated film begins to recover, as expected through the reverse reaction in eqn (1) (Fig. 3A; from 0.566 GΩ to 0.672 GΩ).

## Conclusions

We present, to our knowledge, the first evidence for I_2_ off-gassing, through the solid–air interface, from a mixed-iodide-bromide perovskite following solar illumination. Iodide loss from the bulk perovskite is strongly suggested by a shift to higher optical absorption onset and smaller lattice constant (Fig. 1). By using a solid salt to trap I_2_ vapor directly, we show that the iodide is lost as I_2_, eliminating complications inherent to solid–liquid interfaces (Fig. 2). The effects of heating concomitant with illumination were decoupled through control experiments. In contrast to the effects of heating alone, illumination—and the associated I_2_ off-gassing—substantially increases bulk conductivity through the anticipated n-doping effect (Fig. 3 and eqn (1) forward reaction). Exposing the off-gassed perovskite film to I_2_ vapor decreases its conductivity (Fig. 3 and eqn (1) reverse reaction), evidencing both sides of the I^−^/I_2_ equilibrium.

We propose that irreversible I_2_ loss can masquerade as a “healing” or remixing of a halide-segregated mixed-halide perovskite with illumination. As I_2_ is lost to the atmosphere and the iodide-rich regions of the halide-segregated films are depleted, photogenerated carriers are no longer trapped in these regions. The n-doping that accompanies I_2_ loss may prevent photogenerated charge accumulation in the small-gap iodide-rich regions, further quenching PL from these regions. Thus, the lower-energy PL is lost, to be replaced by the higher-energy PL from photogenerated carriers in the bromide-rich regions, which are more stable to halogen loss due to the higher net positive enthalpy for Br_2_ generation from cleaving Pb–Br bonds. Since the redshifted PL upon illumination has been used as a diagnostic for halide segregation, the loss of this redshifted peak and emergence of the higher-energy PL may only appear as a reversal of halide segregation. Although the prior studies claiming halide-remixing with light^24,25^ used pulsed and continuous-wave lasers at much higher intensities (*e.g.*, 10–200 W cm^−2^) where other dynamics may be at play, we expect that light-soaking with high-intensity light will ultimately degrade the perovskite through I_2_ loss and cannot be used to combat light-induced halide segregation in a device. Our studies were performed at *ca.* 1 sun (0.1 W cm^−2^) continuous illumination and I_2_ loss should be considered as a viable decomposition pathway even at typical solar-cell operating conditions. Although O_2_ may accelerate film degradation under illumination, as shown previously,^26^ O_2_ is not necessary for light-induced I_2_ loss of the illuminated films.

We thus propose the following modified scheme for light-induced halide segregation: For bromide-rich, mixed-halide compositions, the probability of finding a sufficient concentration of surface iodides, capable of forming I–I bonds, is low. For example, if we assume that the bulk halide stoichiometry is maintained at the surface, the probability of finding a [PbI_6_]^4−^ coordination sphere in (MA)Pb(Br_0.75_I_0.25_)_3_ is only 0.02% (Fig. S8†), and likewise the probability of finding two iodides in adjacent octahedra in close enough contact to form I_2_, is also low. However, the local heterogeneity inherent to mixed-halide perovskites^56,57^ leads to a distribution of bromides and iodides at individual Pb centers, with some probability of having coordination spheres of [PbI_6_]^4−^, [PbI_5_Br]^4−^, [PbI_4_Br_2_]^4−^, [PbI_3_Br_3_]^4−^, [PbI_2_Br_4_]^4−^, [PbIBr_5_]^4−^, and [PbBr_6_]^4−^, depending on the overall halide ratio. Thus, we can expect that photogenerated holes will first form at the higher-energy orbitals of iodide-rich Pb centers. Various sophisticated and quantitative models have been proposed to explain the reversible growth of iodide-rich domains under illumination in these materials.^20–22,58,59^ For the purposes of explaining our observations, we simply propose that positive charges (photoexcited holes), which accumulate at the local iodide-rich lead centers, provide an electrostatic driving force for the negatively charged, mobile iodides^31^ in the framework to cluster around these regions, growing the iodide-rich domains and stabilizing the holes. Thus, light-induced phase segregation greatly increases the concentration of neighboring iodides at the surface (Fig. 1A). These iodide-rich domains continue to trap holes and are therefore perfectly poised for I_2_ loss.

Thus, mixed-iodide-bromide perovskites are not protected from I_2_ loss. As we have noted earlier for iodide perovskites,^37^ this further motivates the development of I_2_-impermeable encapsulation materials that can build up the partial pressure of I_2_ at the perovskite surface and drive reaction (1) in reverse through Le Chatelier's principle.

## Note added prior to submission

Just prior to manuscript submission (on 11/28/2024), a study was published online (on 11/25/2024)^55^ reporting I_2_ loss (probed by mass loss and X-ray photoelectron spectroscopy) from mixed-iodide-bromide perovskite films under UV illumination (370 nm; 75 mW cm^−2^) over long durations (24 h) and under vacuum (20 mTorr). Our work shows I_2_ loss from mixed-iodide-bromide perovskite films and powders under simulated solar illumination (AM1.5G through glass to remove the minor UV component; 100 mW cm^−2^) over shorter duration (30 minutes) and at ambient pressure, which are more representative of operating conditions in a solar cell with a standard glass top cover that filters out UV light. The two studies are concurrent, complementary, and completely independent.

## Data availability

Experimental methods, spectra, and the statistical distribution of lead-halide coordination spheres are available in the ESI.†

## Author contributions

The study was conceived by M. L. , J. A. V. and H. I. K. studies on (CH_3_NH_3_)Pb(Br_*x*_I_1−*x*_)_3_ were conducted by M. L. and J. A. V. and studies on CsPb(Br_*x*_I_1−*x*_)_3_ were conducted by Z. J. the manuscript was written by all authors.

## Conflicts of interest

There are no conflicts to declare.

## Supplementary Material

SC-OLF-D4SC08092K-s001
