# Supplementary material for: Evidence for I2 loss from the perovskite–gas interface upon light-induced halide segregation
Source: Chem Sci. 2025 Apr 28;16(22):9662–8. doi: 10.1039/d4sc08092k (PMC12079119; doi:10.1039/d4sc08092k)
Supplement: SC-016-D4SC08092K-s001 [file SC-016-D4SC08092K-s001.pdf]

Supplementary Information (SI) for Chemical Science.
This journal is © The Royal Society of Chemistry 2025

# Supporting Information

# Evidence for $I_2$ Loss from the Perovskite-Gas Interface upon Light-Induced Halide Segregation

Michael Lee,[†] Julian A. Vigil,[†,‡] Zhiqiao Jiang,[†,|] and Hemamala I. Karunadasa[†,∥,*]

[†] Department of Chemistry, Stanford University, Stanford, California 94305, United States
[‡] Department of Chemical Engineering, Stanford University, Stanford, California 94305, United States
[|] Department of Materials Science and Engineering, Stanford University, Stanford, California 94305, United States
[∥] Stanford Institute for Materials and Energy Sciences, SLAC National Laboratory, Menlo Park CA 94025, United States
*e-mail: hemamala@stanford.edu

---

**Table of Contents:**

Supporting Methods ........ S2
Supporting Figures ........ S6
Supporting References ........ S11

**Methods**

The solvents dimethyl sulfoxide (Acros, DMSO), diethyl ether (Sigma-Aldrich, $Et_2O$), and dichloromethane (Alfa Aesar, DCM) were dried and degassed using a JC Meyer solvent purification system, and the solvent γ-butyrolactone (Sigma-Aldrich, GBL) was dried with 3-Å molecular sieves and degassed by three freeze-pump-thaw cycles. All other reagents were purchased from commercial vendors and used as received. Abbreviations used: Ac = acetyl (−$COCH_3$), BYA = but-3-yn-1-aminium, DMF = dimethylformamide, MA = methylammonium ($CH_3NH_3^+$), and MeCN = acetonitrile. BEA-$I_2$ = (*E*)-3,4-diiodobut-3-en-1-aminium, BEA-$Br_2$ = (*E*)-3,4-dibromobut-3-en-1-aminium, BEA-IBr = (*E*)-3-bromo-4-iodobut-3-en-1-aminium, and BEA-HI = 3-iodobut-3-en-1-aminium.

**Mixed-halide perovskite thin film deposition.** Substrates (20 mm × 20 mm glass slides or 15 mm × 20 mm interdigitated indium tin oxide (ITO; 210 nm on glass) substrates) were scrubbed with detergent, sonicated for 10 minutes each in detergent, deionized water, acetone, and isopropanol, dried with compressed air, and treated with a UV-ozone cleaner for 20 minutes. All subsequent manipulations were done under $N_2$.

For (MA)Pb($Br_{0.75}I_{0.25}$)$_3$ thin films, the solid reagents (MA)Br (Greatcell Solar, 33.6 mg, 0.30 mmol), $PbBr_2$ (Alfa Aesar, 110 mg, 0.30 mmol), (MA)I (Greatcell Solar, 15.9 mg, 0.10 mmol), and $PbI_2$ (Sigma-Aldrich, 46.1 mg, 0.10 mmol) were dissolved in a GBL/DMSO mixture (700 μL GBL/300 μL DMSO). The resulting solution was then used immediately after filtration. After 100 μL of the solution was dropped onto a cleaned substrate, the substrate was spun at 1000 rpm for 10 s, then at 5000 rpm for 30 s. An acceleration of 5000 rpm/s was used to reach both speeds. With 15 s remaining in the second stage, 150 μL of $Et_2O$ was quickly dropped onto the film. After annealing at 50 ºC for 5 minutes to remove solvent, the resulting (MA)Pb($Br_{0.75}I_{0.25}$)$_3$ film (halide composition corresponding to the precursor solution) was stored in an $N_2$ glovebox.

For CsPb($Br_{0.75}I_{0.25}$)$_3$ thin films, the spin-coating procedure was modified from a previous report.[1] Solid reagents $PbBr_2$ (183 mg, 0.500 mmol), CsI (Strem, 26.6 mg, 0.125 mmol), and CsBr (Strem, 97.43 mg, 0.375 mmol) were dissolved in 1 mL of DMSO. The solution was stirred at 80 °C until complete dissolution was observed and then used immediately after filtration; the glass substrates were also preheated at 80 °C for 10 minutes. Films were fabricated by spin-coating 200 μL of solution on the hot substrate at 3000 rpm for 1 minute. After annealing at 80 °C for 2 minutes and 200 °C for 10 minutes for solvent removal, the resulting CsPb($Br_{0.75}I_{0.25}$)$_3$ film (halide composition corresponding to the precursor solution) was stored in an $N_2$ glovebox.

**Mixed-halide perovskite powder synthesis.** Vapor diffusion was used to grow $(MA)PbBr_3$ crystals. Solid (MA)Br (112 mg, 1.00 mmol) and $PbBr_2$ (367 mg, 1.00 mmol) were dissolved in DMF (Alfa Aesar, 1 mL) in a 3.7-mL shell vial. The shell vial was sealed in a 20-mL scintillation vial holding 4 mL of DCM, and orange crystals were collected from the shell vial following evaporation of the DCM into the DMF. Solid $(MA)PbBr_3$ (1.25 g, 2.61 mmol) was ground to a fine powder in a mortar and pestle in ambient atmosphere, then dissolved in MeCN (Fisher Scientific, 25 mL) with concentrated aqueous HI (Sigma-Aldrich, 3.75 mL) and HBr (Sigma-Aldrich, 1.25 mL) in a 250-mL round bottom flask. Cold $Et_2O$ (65 mL, 0 °C) was added quickly while stirring, resulting in the precipitation of a red powder. The mixed iodide-bromide perovskite powder was collected and washed with cold $Et_2O$ five times by centrifugation at 4000 rpm for 3 minutes. Excess $Et_2O$ was removed under reduced pressure for 2 h. The composition of the product ($x \sim 0.75$ in $(MA)Pb(Br_xI_{1-x})_3$) was estimated from UV-visible measurement of the band-edge absorption onset (Fig. S2).

**Illumination and heating of thin films.** In a $N_2$ atmosphere, $(MA)Pb(Br_{0.75}I_{0.25})_3$ or $CsPb(Br_{0.75}I_{0.25})_3$ films were sealed in two cylindrical glass chambers (3.5 cm diameter, 7.0 cm height) using Teflon and electrical tape. If the substrate was a 20 mm × 20 mm glass slide, one film was split into two 10 mm × 20 mm slides and sealed in separate chambers. An infinityPV ISOSun solar simulator calibrated to 1 sun intensity (AM1.5G, 0.1 W $cm^{-2}$) was used to illuminate the film through the glass chamber for 30 minutes. The second chamber was heated for 35 minutes in a sand bath, wrapped in foil and maintained at 42 °C, with the additional 5 minutes given to account for heating time. A YSI 55032 thermistor was used to measure the maximum temperature of the chamber under illumination (42 °C; Fig. S3). The films were then rested in the dark for 15 minutes to cool the films to room temperature.

**Illumination and heating of mixed-halide perovskite powders.** Mixed-halide perovskite powder (150 mg) was ground to a finer powder in a mortar and pestle and weighed into a 3.7-mL shell vial. Commercial (BYA)Cl (Chem-Impex, 55 mg) was recrystallized from boiling ethanol (Fisher Scientific, 0.4 mL, 78.5 °C) by the addition of cold $Et_2O$ (2.0 mL, 0 °C) quickly under stirring. The resulting precipitate was collected by filtration and remaining $Et_2O$ was removed under reduced pressure. Recrystallized (BYA)Cl (10 mg) was weighed into a second 3.7-mL shell vial. Under $N_2$ atmosphere, both vials were sealed (with Teflon and electrical tape) in the same chambers as used for the thin-film experiments. This process was repeated to construct a second identical chamber. One chamber was illuminated at 1 sun and the other was heated in a sand bath to 45 °C, both for 15 h.

**X-ray diffraction measurements and fitting.** Diffraction patterns were collected at room temperature (and in ambient atmosphere) on a Bruker D8 Advance diffractometer (Cu $K_\alpha$ radiation, $K_{\alpha,1}$ = 1.54060 Å, $K_{\alpha,2}$ = 1.54443 Å, $K_{\alpha,1}$:$K_{\alpha,2}$ ≈ 2:1; Bragg-Brentano $\theta$-$\theta$ geometry) equipped with fixed divergence slits, a nickel filter, and a LYNXEYE detector. The films were either brought from the glovebox immediately prior to the measurement or transported sealed with desiccant. Nelson-Riley analysis was performed to estimate the true pseudo-cubic lattice parameter, $a$.[2] Peak center positions (in 2θ) for the (100), (200), and (300) pseudo-cubic Bragg reflections (Fig. S4) were obtained by fitting a pseudo-Voigt profile, following a linear baseline subtraction (see example fits in Fig. S5). These centers were linearized against the Nelson-Riley function (NR):

$$\mathrm{NR}(\theta) = \frac{1}{2}\left(\frac{\cos^2\theta}{\sin\theta} + \frac{\cos^2\theta}{\theta}\right)$$

Extrapolation to the back-reflection diffraction condition [$\theta$= 90°, NR($\theta$) = 0] provides the estimation of the true lattice parameter.

**UV-visible spectroscopy measurements of thin films and fitting.** For $(MA)Pb(Br_{0.75}I_{0.25})_3$ thin films, absorption measurements were taken using a Shimadzu UV-2600 spectrometer in transmission mode. The absorption coefficient α was calculated as follows with the optical absorbance, $A$, and the average thickness of the film, $t$ (measured with a Bruker Dektak XT-A profilometer):

$$\alpha = 2.303\frac{A}{t}$$

The optical bandgap was obtained by finding the intersection of the fitted linear baseline and the fitted linear absorption onset when $(\alpha h\upsilon)^2$ is plotted against hυ, where h is Planck's constant and υ the photon frequency.

For $CsPb(Br_{0.75}I_{0.25})_3$ thin films, absorption measurements were taken using a Cary 6000i UV-Vis-NIR spectrophotometer with the diffuse reflectance accessory. The samples were loaded using the center mount with the thin films placed in the middle of the integrating sphere. The pseudo-absorbance spectra were obtained using the Kubelka-Munk transformation and normalized to the peak maxima.[3] The optical bandgap was obtained by finding the intersection of the fitted linear pseudo-absorption onset with the x-axis.

**UV-visible spectroscopy of solvents exposed to mixed halide perovskite powder.** In an $N_2$ atmosphere, mixed-halide perovskite powder (150 mg) was sealed with 4 mL of DCM in a vial fully taped and stored in the dark. After 2 days, the powder was filtered, and absorption measurements of the filtrate were taken.

A second spectrum was taken after dissolving solid $I_2$ in the filtrate to a concentration of $5 \times 10^{-5}$ M. Control spectra were taken after adding (MA)Br and (MA)I to DCM for a concentration of $5.5 \times 10^{-4}$ M and stirring for 1 day. Subsequent spectra were taken after adding $I_2$ and then $Pb(CH_3OO)_2$, both to a concentration of $5 \times 10^{-4}$ M.

**Nuclear magnetic resonance (NMR) spectroscopy measurements.** $^1$H NMR spectra were recorded in fully deuterated dimethyl sulfoxide solution (Cambridge Isotope Laboratories, DMSO-$d_6$) using a 600 MHz Varian Inova NMR spectrometer. The samples [10.0 mg for pure (BYA)Cl] were dissolved in DMSO-$d_6$, filtered, and immediately analyzed by NMR. The obtained spectra were then apodized with a 1 Hz exponential function to remove truncation noise.

**Mass spectrometry measurements.** Mass spectrometry was performed using a Waters Synapt G2-Si LC-MS system with a Waters UPLC BEH C-18 column and an electrospray ionization source. Samples were dissolved in methanol. Due to poor sensitivity in the total ion chromatogram, the single ion chromatographs were tracked for the relevant masses. Exact masses in Da: 70.07 ($BYA^+$), 197.98 ($BEA\text{-}HI^+$), 227.90 ($BEA\text{-}Br_2^+$), 275.89 ($BEA\text{-}IBr^+$) and 323.87 ($BEA\text{-}I_2^+$). Only $BYA^+$ and $BEA\text{-}I_2^+$ were detected in any trials.

**Electrochemical impedance spectroscopy measurements.** Potentiostatic electrochemical impedance spectroscopy (PEIS) measurements were collected using a BioLogic VSP-300 potentiostat in a two-point contact configuration with $(MA)Pb(Br_{0.75}I_{0.25})_3$ films deposited on interdigitated ITO substrates (30 pairs of 80-µm lines, with 80-µm spacing across 8.5-mm fingers). The PEIS measurements were carried out with 0 V DC bias, a sinusoidal driving voltage of 10 mV, and a switching frequency decreasing from 7 MHz to 10 mHz. All measurements were conducted under $N_2$ atmosphere.

## Supporting Figures

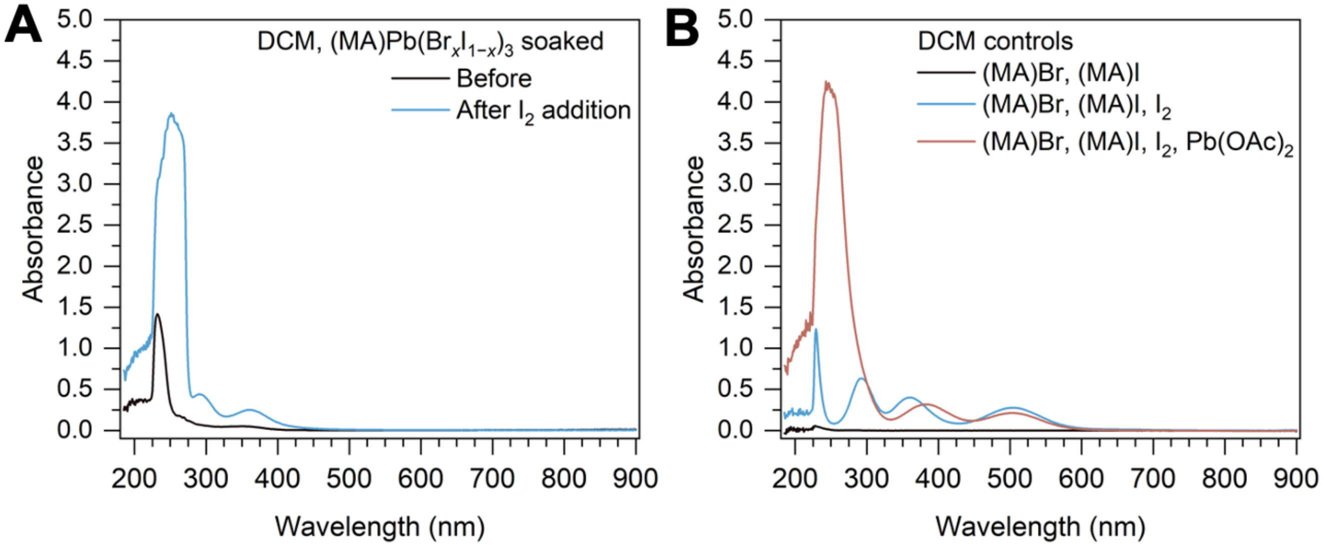


**Figure S1. Dark controls in dichloromethane | (A)** UV-Visible spectroscopy of DCM sealed under $N_2$ for 2 days with $(MA)Pb(Br_xI_{1-x})_3$ powder in the dark before and after $I_2$ addition to the filtrate ($5 \times 10^{-5}$ M). **(B)** UV-Visible spectroscopy of (MA)Br and (MA)I ($5.5 \times 10^{-4}$ M), $I_2$ ($5.5 \times 10^{-4}$ M) and $Pb(OAc)_2$ ($5.5 \times 10^{-4}$ M) in DCM.

---

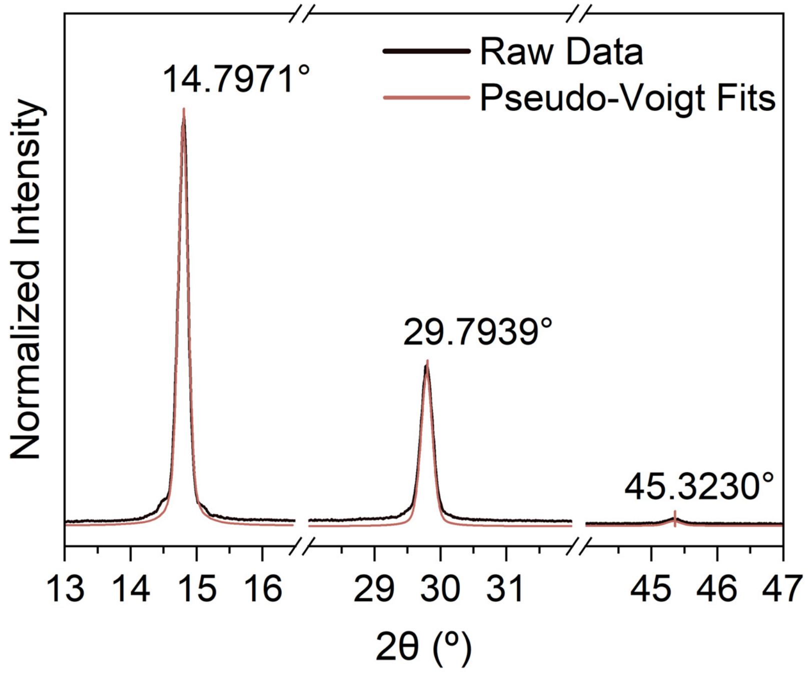


**Figure S2. Example peak fits for Nelson-Riley analysis |** Pseudo-Voigt fits of pseudo-cubic ($h$00) ($h$ = 1, 2, 3 with increasing 2θ) reflections of the synthesized $(MA)Pb(Br_{0.75}I_{0.25})_3$ thin film; labels denote the fitted peak centers (in deg. 2θ).

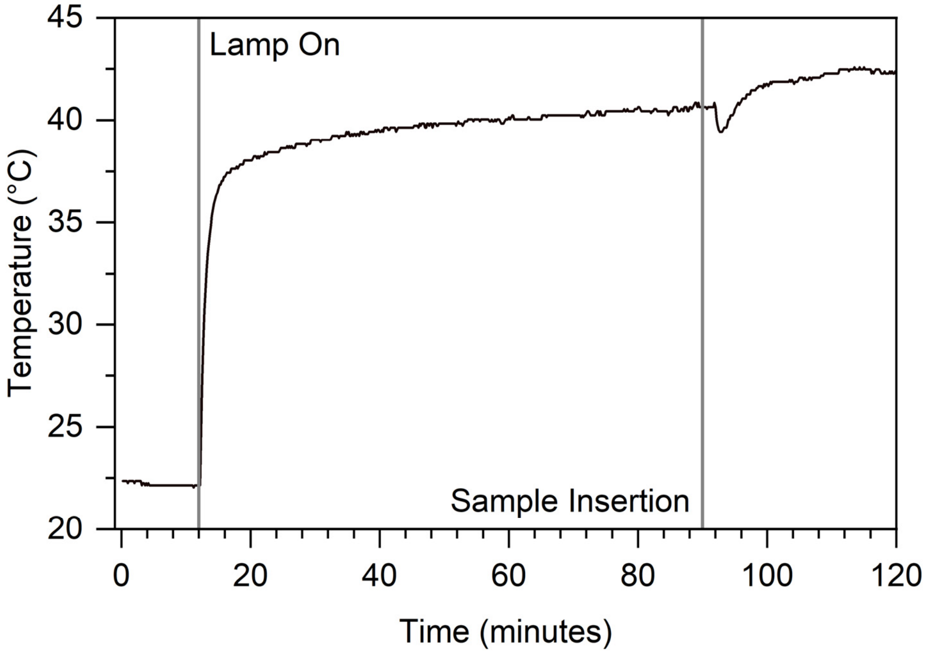


**Figure S3. Temperature during illumination trial |** Temperature of the chamber base measured with a thermistor. The lamp was turned on at 12 minutes and the film placed on the surface at 90 minutes.

---

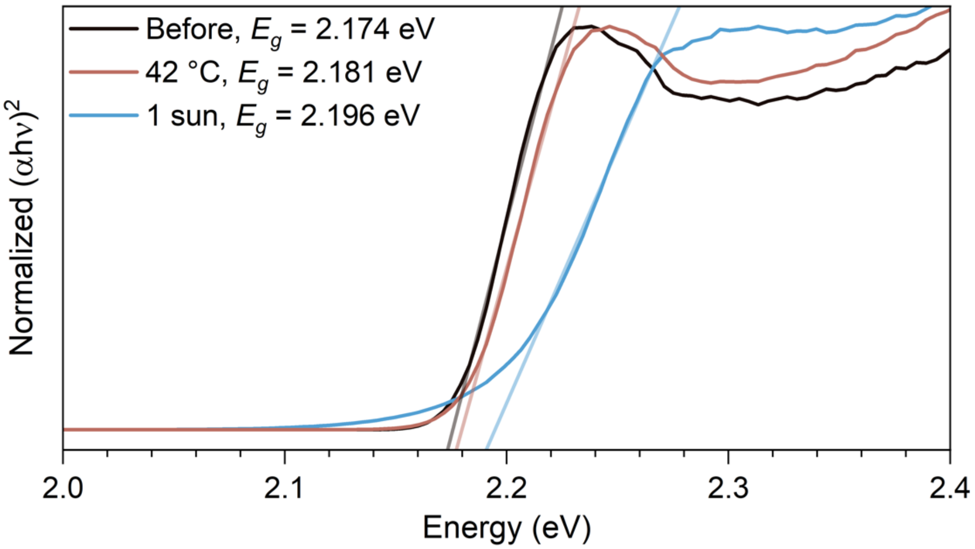


**Figure S4**. **UV-vis absorption spectra for $CsPb(Br_{0.75}I_{0.25})_3$ thin films** | Tauc plots from Kubelka-Munk pseudo-absorbance of $CsPb(Br_{0.75}I_{0.25})_3$ thin films following heating at ca. 42 °C (35 minutes) or illumination at 1 sun (30 minutes).

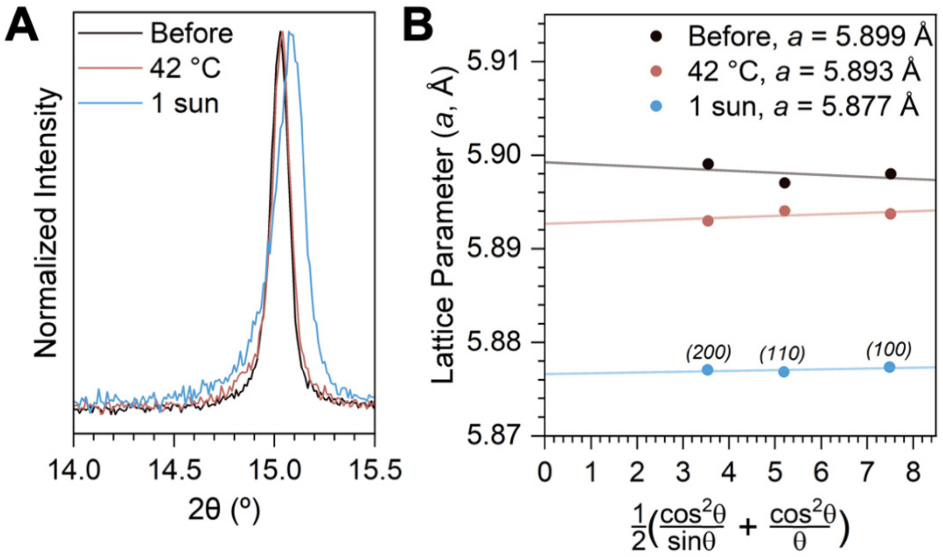


**Figure S5**. **X-ray diffraction patterns for $CsPb(Br_{0.75}I_{0.25})_3$ thin films** | **(A)** X-ray diffraction patterns showing the shift in the pseudo-cubic (100) Bragg reflection of the $CsPb(Br_{0.75}I_{0.25})_3$ thin films following heating or illumination. **(B)** Pseudo-cubic lattice parameter, *a*, extracted using the Nelson-Riley method (see Methods).

---

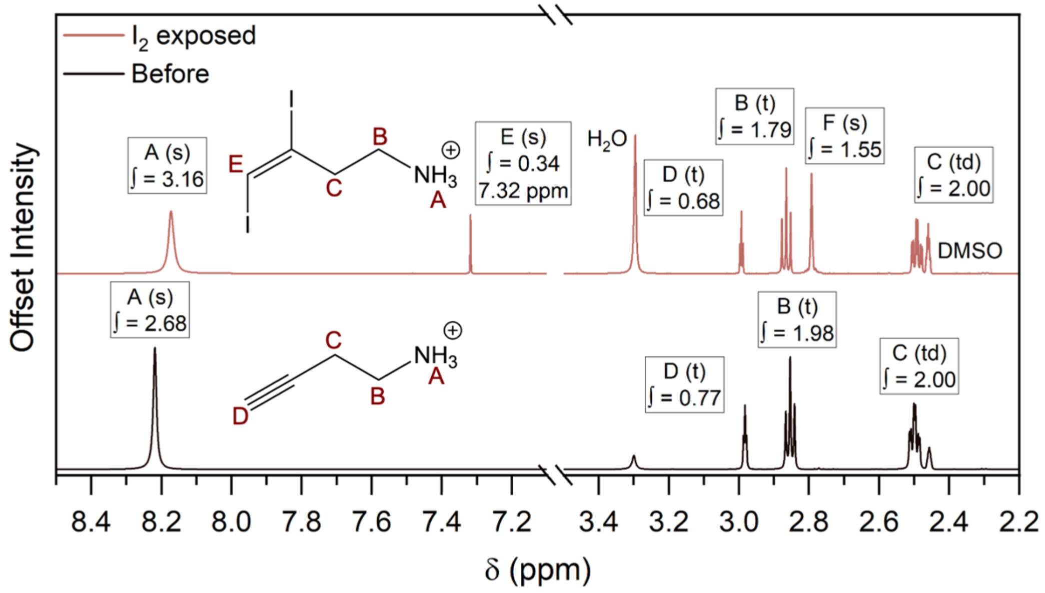


**Figure S6. $^1$H NMR controls** | $^1$H NMR spectra of $BYA^+$ before and after exposure to a saturated $I_2$ atmosphere for 16 h. The peak labeled "F" is tentatively assigned to a DMS-$I_2$ complex due to the known reactivity between $I_2$ and DMSO at high $I_2$ concentrations.[4,5]

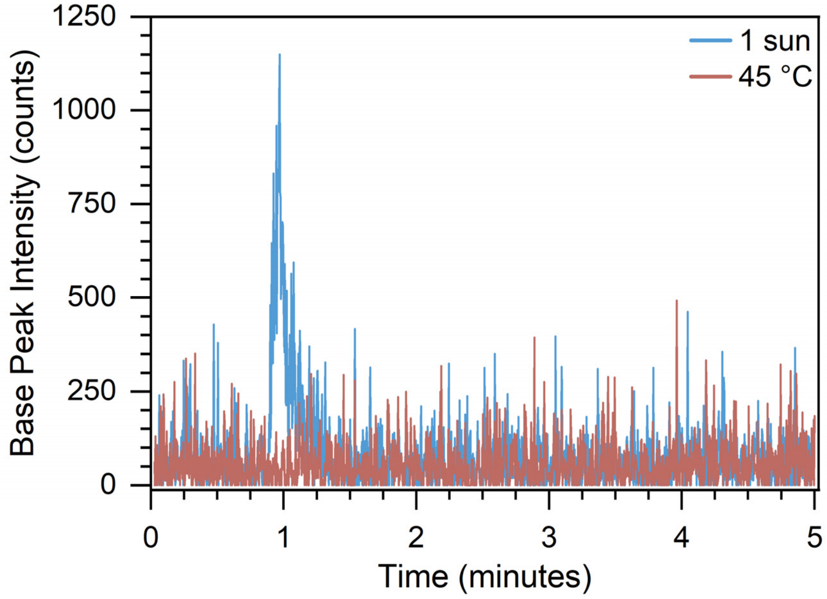


**Figure S7. Targeted-ion chromatogram of (BEA-$I_2$) |** Targeted-ion chromatogram ($m/z$ = 323.875) from liquid chromatography of (BYA)Cl following illumination or heating in the presence of (MA)Pb($Br_xI_{1-x}$)$_3$ perovskite powder.

---

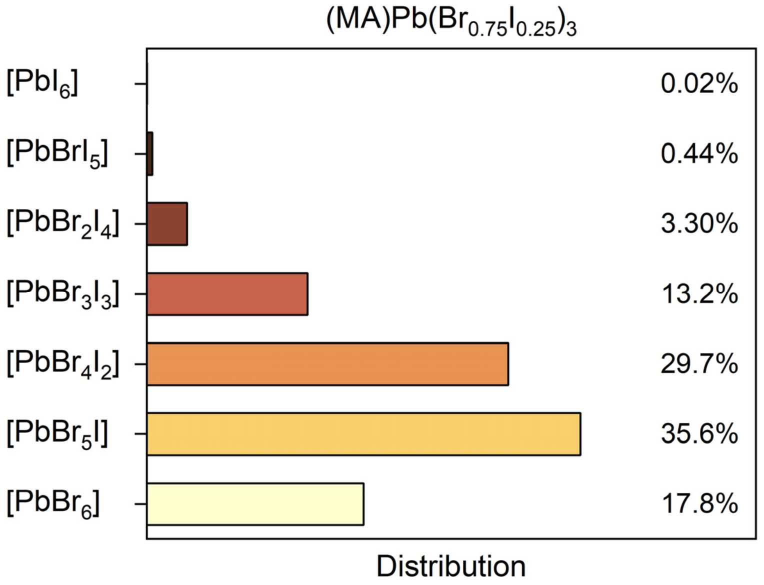


**Figure S8. Statistical distribution of lead coordination spheres |** Percentage abundance of [$PbX_6$] octahedra in (MA)Pb($Br_xI_{1-x}$)$_3$ ($x$ = 0.75), assuming a binomial distribution.

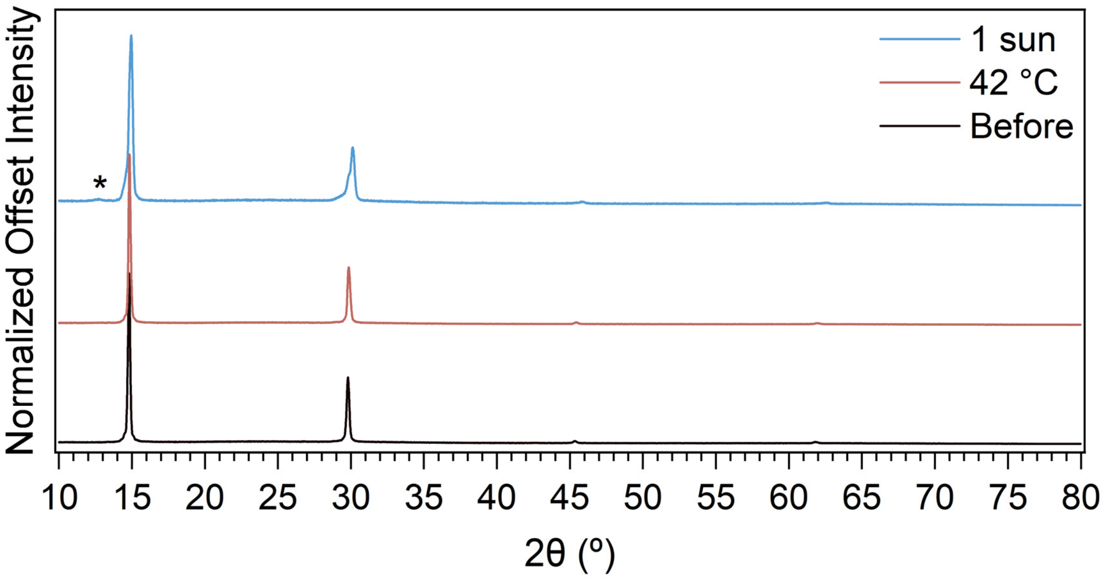


**Figure S9. XRD patterns of (MA)Pb($Br_xI_{1-x}$)$_3$ films |** Full 2θ scan range of the X-ray diffraction (XRD) patterns appearing in the main text, Fig. 1C. The small feature marked with an asterisk matches the (001) reflection of a Pb($Br_xI_{1-x}$)$_2$ mixed halide isostructural to $PbI_2$.

---

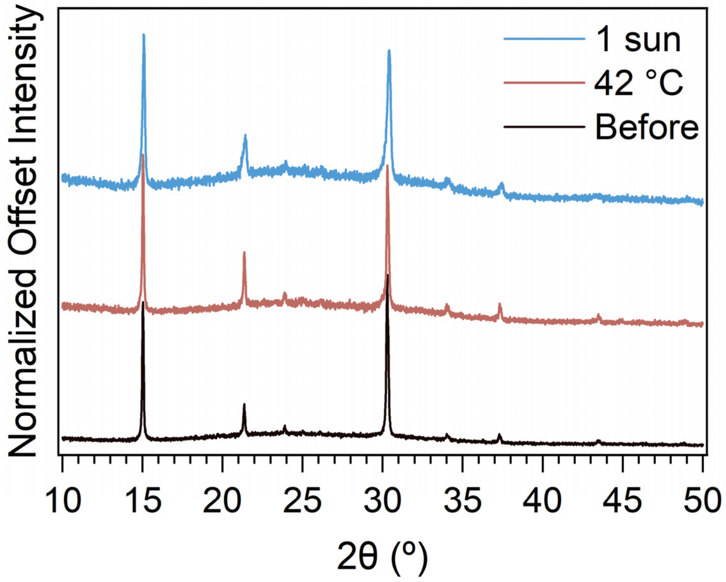


**Figure S10. XRD patterns of CsPb($Br_xI_{1-x}$)$_3$ films |** Full 2θ scan range of the X-ray diffraction (XRD) patterns appearing in Supplementary Information Figure S6. The overall powder pattern can be fitted as pseudo-cubic with the *Pm-3m* space group. The three small features between 24-26° (i.e., at 23.9°, 25.0°, and 26.1° for the "before" sample) can still be indexed to CsPb($Br_xI_{1-x}$)$_3$ but in lower-symmetry space groups (tetragonal *P/4mbm* or orthorhombic *Pnma*).

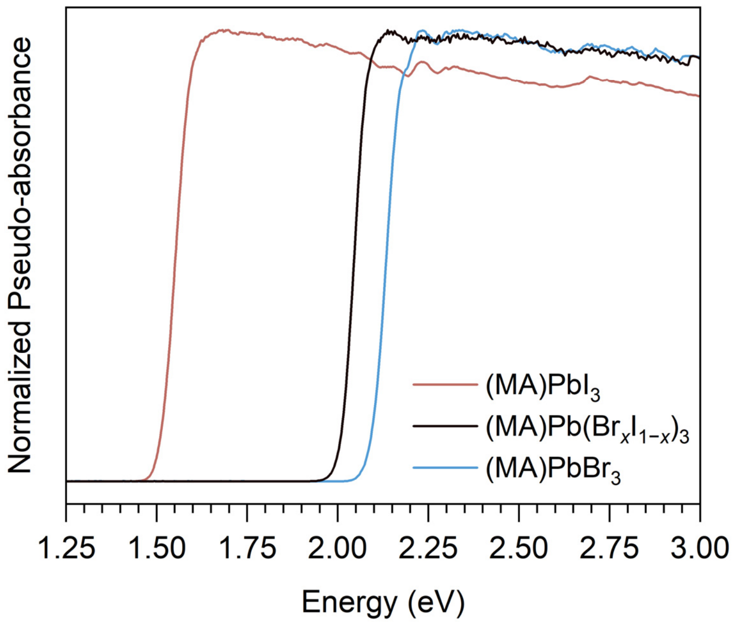


**Figure S11. UV-visible absorption spectra of halide perovskite powders |** Kubelka-Munk pseudo-absorbance of the synthesized (MA)Pb($Br_xI_{1-x}$)$_3$ powder compared with those of the endmembers: (MA)$PbBr_3$ and (MA)$PbI_3$.

## Supporting References

1 V. O. Eze, L. B. Carani, H. Majumder, M. J. Uddin and O. I. Okoli, *Sci Rep*, 2022, **12**, 7794.
2 J. B. Nelson and D. P. Riley, *Proceedings of the Physical Society*, 1945, **57**, 160–177.
3 P. Kubelka and F. Z. Munk, *Z. Tech. Phys.*, 1931, **12**, 593–601.
4 R. Reetu, S. Kalita, S. Dash and C. C. Malakar, *ChemistrySelect*, 2024, **9**, e202303845.
5 A. Monga, S. Bagchi and A. Sharma, *New J. Chem.*, 2018, **42**, 1551–1576.
